# Data Mining of Polymer Phase Transitions upon Temperature Changes by Small and Wide-Angle X-ray Scattering Combined with Raman Spectroscopy

**DOI:** 10.3390/polym13234203

**Published:** 2021-11-30

**Authors:** Sarah Saidi, Giuseppe Portale, Wim Bras, Alessandro Longo, José Manuel Amigo, David Chapron, Patrice Bourson, Daniel Hermida-Merino

**Affiliations:** 1 Laboratoire Matériaux Optiques, Photonique et Systèmes (LMOPS), Université de Lorraine, CentraleSupelec, 2 Rue Edouard Belin, 57070 Metz, France; saidi.sarah1@hotmail.fr (S.S.); david.chapron@univ-lorraine.fr (D.C.); patrice.bourson@univ-lorraine.fr (P.B.); 2 Netherlands Organization for Scientific Research (NWO), DUBBLE@ESRF, BP CS40220, 38043 Grenoble, France; brasw@ornl.gov; 3 Macromolecular Chemistry & New Polymeric Materials, Zernike Institute for Advanced Materials, University of Groningen, Nijenborgh 4, 9747 AG Groningen, The Netherlands; g.portale@rug.nl; 4 Oak Ridge National Laboratory, Chemistry Division, Oak Ridge, TN 37831, USA; 5 European Synchrotron Radiation Facility (ESRF), 71 Avenue des Martyrs, 38000 Grenoble, France; alessandro.longo@esrf.fr; 6 Istituto per lo Studio dei Materiali Nanostrutturati (ISMN)-CNR, UOS Palermo, Via Ugo la Malfa, 153, 90146 Palermo, Italy; 7 IKERBASQUE, Basque Foundation for Science, 48011 Bilbao, Spain; josemanuel.amigo@ehu.eus; 8 Department of Analytical Chemistry, University of the Basque Country UPV/EHU, 48080 Bilbao, Spain

**Keywords:** polymer, X-ray scattering, Raman spectroscopy, combined techniques, multivariate analysis

## Abstract

The complex physical transformations of polymers upon external thermodynamic changes are related to the molecular length of the polymer and its associated multifaceted energetic balance. The understanding of subtle transitions or multistep phase transformation requires real-time phenomenological studies using a multi-technique approach that covers several length-scales and chemical states. A combination of X-ray scattering techniques with Raman spectroscopy and Differential Scanning Calorimetry was conducted to correlate the structural changes from the conformational chain to the polymer crystal and mesoscale organization. Current research applications and the experimental combination of Raman spectroscopy with simultaneous SAXS/WAXS measurements coupled to a DSC is discussed. In particular, we show that in order to obtain the maximum benefit from simultaneously obtained high-quality data sets from different techniques, one should look beyond traditional analysis techniques and instead apply multivariate analysis. Data mining strategies can be applied to develop methods to control polymer processing in an industrial context. Crystallization studies of a PVDF blend with a fluoroelastomer, known to feature complex phase transitions, were used to validate the combined approach and further analyzed by MVA.

## 1. Introduction

Time-resolved small-angle X-ray scattering (SAXS) and wide-angle X-ray scattering (WAXS) are [1] routine structural characterization techniques in the synchrotron radiation community. Furthermore, through a combination of improvements in X-ray generators, optics and detectors, SAXS laboratory experiments have entered the time-resolved domain. With these technical advances, a novel approach to the analysis of complex data sets might be required to reap the full benefits of such a multimodal approach.

At present, there is a significant number of multi-purpose synchrotron radiation SAXS/WAXS beamlines, in which a plethora of materials science experiments in combination with sophisticated sample environments can be carried out to monitor the nanostructure development upon external stimuli [2,3,4,5].

In particular, polymer science under extreme conditions has been largely studied by simultaneous SAXS/WAXS experiments [6] in combination with sophisticated sample environments that reproduce specific processing thermodynamic conditions or replicate pilot plants in the manufacturing field [7]. The emphasis on combined techniques has been proven to be very beneficial for the elucidation of polymer nanostructure formation (mainly crystallization) under processing-relevant conditions that mimic industrial environments, such as fast quenching [8,9], solid-state films drawability [10], 2D stretching [10], thin film formation by spin coating [11,12,13] or polymerization under sc-CO_2_ [14,15,16,17] and, recently, 3D printing [18,19] and laser sintering [20]. Substantial efforts have been dedicated to the understanding of the interplay of the different flow fields [21,22,23,24,25,26] and thermal treatments [1] that replicate the industrial conditions at which polymers are subjected in processing, such as extrusion, injection moulding [27], blow moulding [28,29], fibre spinning, drop-casting, filament deposition modelling and inkjet printing on crystallization.

Experimental strategies to perform simultaneous SAXS-WAXS experiments in combination with non-X-ray based techniques with different sensitivities have been extensively discussed [30], as well as the design of appropriate sample environments. The simultaneous multiple techniques approach offers the major advantage that there are no time- or thermodynamic condition shifts (thermal herein) between the different data sets and, thus, identical kinetics are monitored by each technique when influenced by the X-ray interaction. In particular, the combination of vibrational spectroscopy technics (FTIR, Raman Scattering) with X-ray scattering [9,31] and more recent work using SAXS, Ultraviolet-visible (UV-Vis) [32], as well as Raman spectroscopy [33], extends the structural analysis by probing the chemical environment and its fundamental arrangement nature (interactions, chain conformation, secondary structure). Likewise, the combination of scattering techniques with vibrational techniques enables to cover the structural transformations at different length scales and to establish the driving force between the morphological transitions and the chemical conversions.

However, the technical requirements for conducting the widely used Fourier-transform infrared spectroscopy (FTIR) hamper the combination with other techniques and specific complex sample environments have to be designed to be coupled for each experiment requirement. By contrast, Raman spectroscopy has been widely used in combination with multiple techniques, in particular for polymer characterization in the industrial field [34,35] such as monitoring of in situ polymerization by Raman spectroscopy [36,37,38] as well as rheo-Raman [39] and Raman-DSC [40], due to its simple implementation, improvement of the time resolution and experimental advantages [41]. Furthermore, Raman spectroscopy is a non-destructive technique that can be applied to different media (bulk, fibres, solution, gels and films), specifically aqueous solution without special experimental preparations, and requires small quantities of sample to be studied [41,42]. In addition, Raman spectroscopy is a key complementary technique to X-ray scattering as Raman scattering probes the covalent nature of the polymeric backbone, particularly the frequent unsaturated bonds as well as the chain stereoregularity [43]. Besides, the technical ease to perform polarized Raman spectroscopy, the scattering nature of the Raman spectroscopy enables it to be sensible to phase transitions related to the laser wavelength, thus extending the length scale range under investigation. In addition, the low-frequency spectral range correlates with the collective motion associated with the transition time to identify and relate the chemical conformation to the structure of nanomaterials, which is especially beneficial for polymer characterization. Moreover, the detailed description of the nanostructure composition by SAXS experiments benefits from correlations with quantitative Raman analysis to optimize the parameters of the SAXS-derived correlation function, particularly at crystallinities around 50%, where electronic contrasts hamper the phase assignment by SAXS. In particular, Raman spectroscopy has been coupled to several X-ray-based techniques across the synchrotron community for both scattering areas, such as micro-X-ray Diffraction (XRD) [44], high resolution [45] XRD or spectroscopic techniques, including UV-vis X-ray absorption fine structure (XAFS) [46] and sequential combinations of both as X-ray absorption near edge spectroscopy (XANES)–XRD [47]. Moreover, Raman spectroscopy is beneficially associated with SAXS-WAXS techniques to analyze the nano-structural evolution of polymeric systems upon external stimuli while obtaining concurrently kinetic data, as well as identifying potential X-ray beam damage [48,49]. Simultaneous Raman spectroscopy with high time resolution (2 s) on backscattering mode using an optical fiber probe with time-resolved SAXS-WAXS measurements [31] has been applied to follow in situ the phase transition of high-density polyethylene upon thermal treatment, in combination with a Linkam calorimeter stage [50] that has been recently expanded to X-ray lab machines [51]. However, the quality of the spectroscopy data due to experimental limitations, as well as the interpretation of the large amount of data generated, has restricted its routine use.

In addition to experimental issues such as differences in the required acquisition time between the different techniques, there is also the challenge of obtaining the sometimes subtle correlations between the different data sets to correlate these with structural changes.

The correlation of the variables of a complex system that define the structural transformations obtained from multiple techniques [52], particularly in this study, which used WAXS, SAXS and Raman spectroscopy as a function of temperature, is crucial to relate ambiguous or/and unclear correlations among the crystallinity (Xc), long period (Lp), conformational changes and phase transition of a dynamic structural evolution upon cooling. Generally, data analysis so far has focused on an area of interest of the spectra, in which evident changes are observed and compared to other techniques data. However, as we show in this study, data sets can contain less obvious cross-correlations that are difficult to perceive. Raman spectra analysis typically consists of the decomposition to a Voigt function [47,48] of the Raman band (Lorentz-Gaussian outline) pre-processed by correcting the raw data with a baseline, smoothing and then normalizing the spectra to an independent band of the process under study. However, subjectivity introduced into the analysis on the normalization and deconvolution inaccuracy of superimposed Raman bands generally involves data treatment and previous knowledge about the Raman modes.

Chemometrics [53] uses statistical methods, such as Multivariate analysis (MVA), to study complex sets of chemical data that contain more than one variable, particularly by using Principal Component Analysis (PCA). MVA and PCA can extract additional information from data, especially in the case of complicated phenomena and/or small changes. MVA has found widespread use in spectroscopy data analysis. [54] However, so far, it has only been applied in a few instances in combination with X-ray scattering techniques such as inverse gas chromatographic [55], SAXS/UV-vis [33,56], SAXS and high-performance anion-exchange chromatography using pulsed amperometric detection (HPAEC-PAD) [57]. Furthermore, chemometrics models have been exploited to analyze polymer crystallization transitions under processing conditions [58].

Before applying MVA methods, the spectral data pre-treatment to correct the experimental artefacts has been shown to be important and was [59] evaluated for different material states (melt state to semi-crystalline solid state) and polymer postprocessing procedures (cooling down, heating up, etc.) in the applied chemometrics model (PCA, partial least squares (PLS)).

The choice of a suitable Raman spectral range of interest to apply the chemometric model is crucial to observe small but important variations that otherwise go unnoticed if the data sets are analyzed separately. In particular, the crystallization mechanism at quiescent conditions of molten poly(3-hydroxybutyrate) (PHB) has been analyzed in detail by Multivariate Curve Resolution (MCR), as well as both homospectral (both spectra from the same technique) and heterospectral (spectra from different techniques) Two Dimension Correlation Spectroscopy (2DCOS), to reveal the sequential order of the multistep crystallization process [60]. However, the scrutiny of multiple (more than two) datasets by data fusion strategies in polymer crystallization offers the possibility to understand the hidden exchanges and time correlations between different complementary techniques probing the same phenomena.

Herein, we describe a simultaneous time-resolved SAXS/WAXS experimental set-up in combination with both Differential Scanning Calorimetry (DSC) as well as Raman spectroscopy that renders information-rich data sets that allow MVA techniques to be applied. The versatile designed set-up features accurate temperature control; the different techniques were controlled by a single data acquisition system generated a multiple dataset that was grouped by data fusion approaches, and further analyzed by MVA.

The chemometrics models were applied to the data fusion database to enrich the analysis of the single techniques by uncovering the correlations between the different techniques variable components. This enables to retrieve slight data variance and recognize unclear variable assignments and their time evolutions.

Multi-technique simultaneous analysis was applied to the complex crystallization mechanism upon cooling from the melt to a fluoroelatomer/PVDF blend to reveal the hidden correlations between weak signals of controversial PVDF transitions in previous studies. In particular, the multidataset combined analysis helped us to learn the identification of the barely discernible Raman vibration modes of both the amorphous and crystalline PVDF phases, which, in turn, were used to associate the higher-sensibility Raman components at the faint transitions.

## 2. Experimental Section

### 2.1. Materials

All the materials were supplied by Arkema and used without further treatment. The PVDF homopolymer featured a melt flow index (MFI) of 2 g/10 min at 230 °C. The fluoroelastomer consisted of vinylidene fluoride-hexafluoropropylene (VF_2_/HFP) copolymer, with a melt viscosity of 4000 Pa·s at 230 °C under 100 s^−1^. The PVDF/fluoroelastomer blend (25 weight per cent) was blended in the melt by extrusion. The samples were supplied as 4 mm thick compression molded sheets. Holes were punched in the DSC pans and subsequently covered with mica windows to allow the Raman laser and scattering to pass through the DSC sample pans.

### 2.2. Raman Spectroscopy Combined with Simultaneous SAXS/WAXS Coupled to DSC

Time-resolved simultaneous SAXS and WAXS experiments (DUBBLE, BM26, at European Synchrotron Radiation Facility, Grenoble, France) coupled to a Linkam calorimeter (LINKAM SCIENTIFIC INSTRUMENTS LTD, Tadworth, United Kingdom)stage have become a routine technique in the synchrotron light community; however, herein a detailed description of the experimental alternatives is presented in order to benefit from the advantages of the data acquired by in situ Raman spectroscopy.

**Figure 1:**
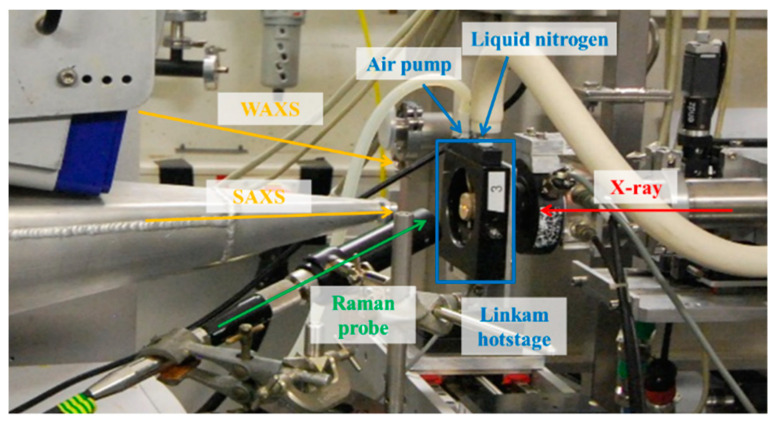
The simultaneous SAXS/WAXS combined with both Linkam DSC and Raman spectroscopy setup at the Dutch-Belgian Beamline (DUBBLE, BM26) at the European Synchrotron Radiation Facility (ESRF).

Raman scattering (RXN1 spectrometer from Kaiser Optical Systems, Ann Arbor, MI, USA) was conducted in the reflection mode fitted with focusing optics by placing the probe under an angle to avoid blocking the X-rays and to minimize air scattering (Figure 1) while the DSC stage allowed X-ray scattering in transmission mode.

The synchronization of the different techniques was achieved using a Multipurpose Unit for Synchronization, Sequencing and Triggering (MUSST) electronic module as a central unit. The MUSST unit is fitted with an internal timer that is independent and flexible for triggering and synchronizing different beamline components and user hardware. The MUSST built-in data storage capacity can be used as a general data acquisition unit for combined techniques. Moreover, the MUSST unit offers asynchronous periodic polling over an extended period. In a typical experiment, a configuration file with the thermal protocol has to be created to send synchronization triggers to the different detector systems [61]. The system writes the corresponding thermal data, as well as a file containing the DSC data to avoid temporal mismatches, into the header of each SAXS/WAXS frame.

The experimental procedure allows the future inclusion of additional sample environments or auxiliary experimental techniques.

The Raman laser wavelength and power must be selected depending on the sample characteristic to enhance the response while minimizing sample damage. The Raman spectrometer used allows the selection of laser wavelengths that enables to tune the Raman response (532 nm lasers for higher sensitivity or resonance enhancement, 633 nm lasers for efficiency and low fluorescence, 785 nm lasers for sample fluorescence suppression) as well as the laser power (100–400 mW).

**Figure 2:**
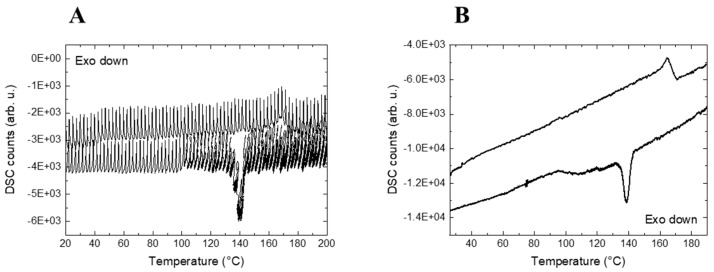
Linkam DSC profile of the cooling and second heating ramps of the PVDF sample during the simultaneous SAXS/WAXS coupled to the Linkam DSC combined with the Raman spectrometer with the activated laser shutter (**A**) and with the deactivated laser shutter (**B**).

For our experiments, a 785 nm laser wavelength with a 150 mW output was selected to minimize the material fluorescence and laser heat transfer. The positioning of the laser beam focus on the sample also enables to control the illuminated sample volume, as well as the laser light penetration (Gaussian-shaped beam), depending on the experiment requirements. The selected focal distance of 5 cm corresponds to a focal point waist dimension [62] of 2*W*_0_ = 20 µm and 2*Z_R_* = 2.1 mm, obtained by the following equation:

(1) $$2W_{0}=\frac{4\lambda f}{\pi \phi }$$

(2) $$2Z_{R}=\frac{16\lambda nf2}{\pi \phi 2}$$

with $W_{0}$ as the diameter of the convergent lens, *λ* as the wavelength of the laser (785 nm), *f* as the focal distance (5 cm), *ϕ* as the output laser beam diameter (2.6 mm) [63,64], $Z_{R}$ as the convergent lens distance and *n* as the refractive index (1.42 for neat PVDF, 1.30 for HFP and ca 1.40 for the 75/25% PVDF/VF_2_/HFP blend). The sample thickness was 0.8 mm. In addition, the absorption of laser light induces sample heating (photothermal effect) when the energetic excitation rate of the material is slower than the thermalization time [65]. Typically, polymers are characterized in the lower part of the thermalization times, in the order of 10^−6^ s. Heating due to X-ray absorption was negligible in our experiments, as well as the radiation damage produced by the X-ray doses that we used [66,67]. The incident laser beam of our spectrometer was synchronized with the shutter aperture to obtain a better signal/noise ratio by avoiding parasitical light. However, an oscillating Linkam DSC signal with a frequency associated with the opening laser shutter period was observed (see Figure 2A) due to the laser intermittently impinging on the sample while the deactivation of the laser shutter yielded a constant Linkam DSC signal (see Figure 2B); and thus, it was kept open during our experiments.

The calibration of the sample temperature in the coupled techniques experiment was conducted (see Figure S1; the figures designed as SX are available in the supplementary material document) to correct both the X-ray [67] and laser beam (785 nm) effects on the DSC thermocouple reading. The maximum deviation of the set temperature was 2 °C.

The quality of the in situ Raman spectra acquired in this combined experiment was compared with the data obtained by a standard standalone DSC combined with a Raman spectrometer (see Figure S2A) [68] to evaluate the adequacy of the Raman spectra to assign transitions. Similar spectral data quality was obtained in both Raman setups, apart from the smoother profile recorded in the DSC standalone Raman spectra due to the lower background (see Figure S2B).

**Figure 3:**
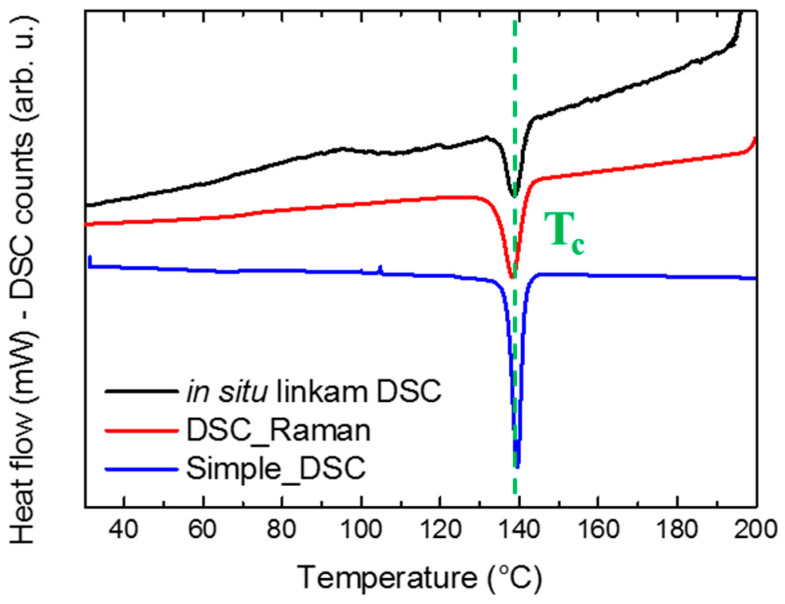
Comparison of the thermograms obtained by the Linkam DSC600 setup (X-rays and Raman laser on), the Q200 TA DSC-Raman setup (Figure S2A available in the supplementary material document) and a single Q200 TA DSC profile. Slight discrepancies in the crystallization temperature were observed, as well as a slight drift in the baseline.

A thermal protocol with identical ramp parameters was applied to the PVDF/VF_2_-HFP blend by using three different experimental setups to validate the temperature acquisition routine. The temperature reading of the in situ Linkam DSC combined with both Raman spectroscopy and X-ray scattering techniques, with the combined DSC standalone-Raman using a Q200 TA Instrument (New Castle, DE, USA) [68] and the Q200 TA Instrument DSC as a reference, were compared. A slight temperature deviation was found even after correction for laser and beam thermal influence (see Figure 3) for the crystallization temperature (138.7 °C, 138.1 °C and 139.5 °C, respectively, for in situ Linkam DSC, DSC_Raman and Simple_DSC). The temperature differences between the three crystallization temperatures were systematic and not statistical errors.

### 2.3. Multivariate Analysis

The multivariate data analysis was conducted using PLS_Toolbox (version 8.8. Eigenvector Research, MA, USA) to perform the SUM-PCA [69] and the MCR-ALS toolbox, available at (http://www.mcrals.info/, 28 November 2021) [70], was used to perform the multiblock-MCR. Both toolboxes worked under Matlab environment (version 2019b, The Mathworks, Natick, MA, USA). A detailed description of the methodology applied is available in the Supplementary Materials (Section S2 in the Supplementary Materials).

## 3. Results and Discussion

### 3.1. Combined Analysis

The crystallization PVDF mechanism involved several polymorphic semi-crystalline structures (namely α (TGTG’), β (TTTT), and γ (TTTGTTTG’) phases). Therefore, it was selected as the candidate to highlight the impact of the crystallization conditions in the final nanostructure by chemometric analysis of the multiprobe-acquired data. PVDF at moderate quenching temperatures crystallizes from the melt, mainly in the α phase, while high quenching temperatures, blending with other polymers such as PMMA [71,72], PA6 [73] and ionic liquid [74], or stretching forces are required to obtain the β phase (which is electroactive) [75,76].

The multi-technique approach to monitoring the PVDF/fluoroelastomer blend crystallization can resolve several ambiguous assignments that would otherwise result from applying a single technique. For instance, the β-phase quantification of PVDF at low contents is difficult to quantify by WAXS alone due to the broad profile of the reflections and the superposition with the amorphous phase. Likewise, Raman spectroscopy cannot rigorously quantify and identify the amorphous phase due to the overlap with the α-phase (the amorphous phase of PVDF is convoluted with the band of the α-phase at 796 cm^−1^) but is very sensitive to the β phase instead.

**Figure 4:**
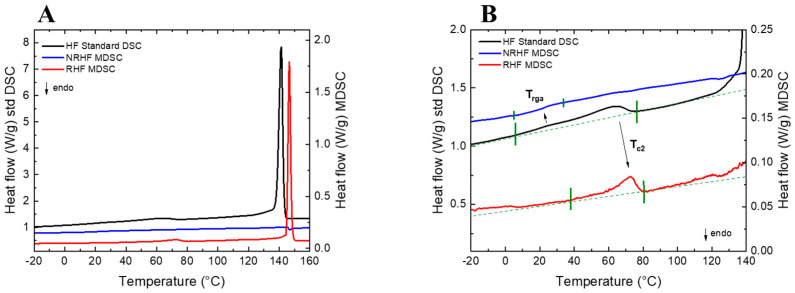
Thermal transitions obtained by standard DSC and MDSC upon cooling from the Melt (**A**), and zoom in of DSC/MDSC thermogram corresponding to the controversial weak thermal transitions of the rigid amorphous phase densification (T_rga_) and of a secondary crystallization (T_c2_) (**B**).

Similarly, the crystallization mechanism from the melt (200 °C) of PVDF is known to be complex. In particular, the wide crystallization temperature range of the PVDF/fluoroelastomer blend shown by Modulated Differential Scanning Calorimetry (MDSC), where, after a fast-narrow endotherm, an asymmetric upward return extended for a large temperature range with a monotonous low energetic profile was evident, suggests a continuous crystallization process that is invisible by standard DSC (Figure 4A,B).

Furthermore, the effect of the addition of fluoroelastomer on the crystallization of PVDF has not yet been investigated. The typical head-to-tail configuration of PVDF, as well as the related head-to-head and tail-to-tail chain defects, have been identified to allow complex nanostructures identifiable by weak thermal transitions and different chain mobilities domains. Moreover, upon further cooling, a subsequent weak structural transition was observed at 70 °C; that was previously controversially assigned [77,78,79,80] either to the rigid amorphous phase densification (T_rga_) or the onset of a secondary crystallization (T_c2_) (B) (Figure 4B).

**Figure 5:**
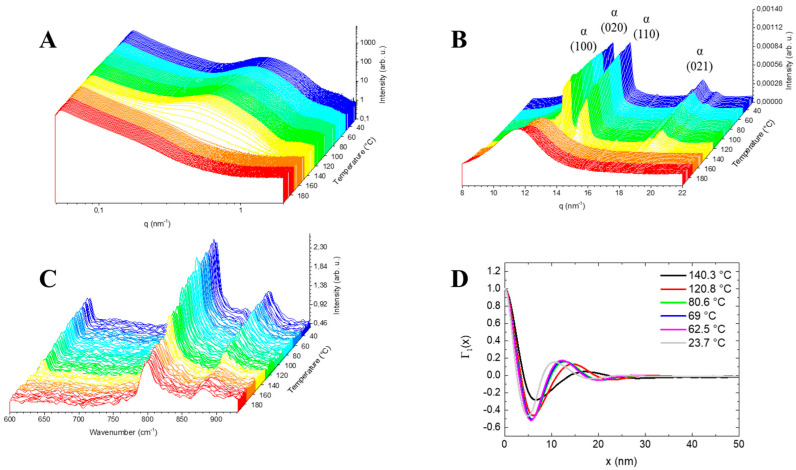
Three-dimensional plots of the time-resolved SAXS (**A**) and WAXS (**B**) pattern (100), (020), (110) and (021) (hkl) planes of the monoclinic PVDF phase (pseudo-orthorhombic) [81] and Raman spectra (**C**), obtained using in situ Raman spectroscopy combined with SAXS/WAXS coupled to a Linkam DSC setup, during the cooling step from the melt to room temperature (RT) of the blend PVDF/fluoroelastomer sample. (**D**) Correlation function derived from the SAXS data of the PVDF blend derivative upon cooling.

The detailed structural analysis, long period evolution and degree of crystallinity were monitored simultaneously by SAXS (Figure 5A) and WAXS (Figure 5B) respectively. The growth of the WAXS reflections showed the transformation from the amorphous to the α-PVDF phase.

The absence of diffraction peaks associated with the β-phase during crystallization could have resulted from either the low phase sensitivity or the crystallinity threshold detection limit of the experimental technique (WAXS), which is 1–3% in polymer study [82].

**Figure 6:**
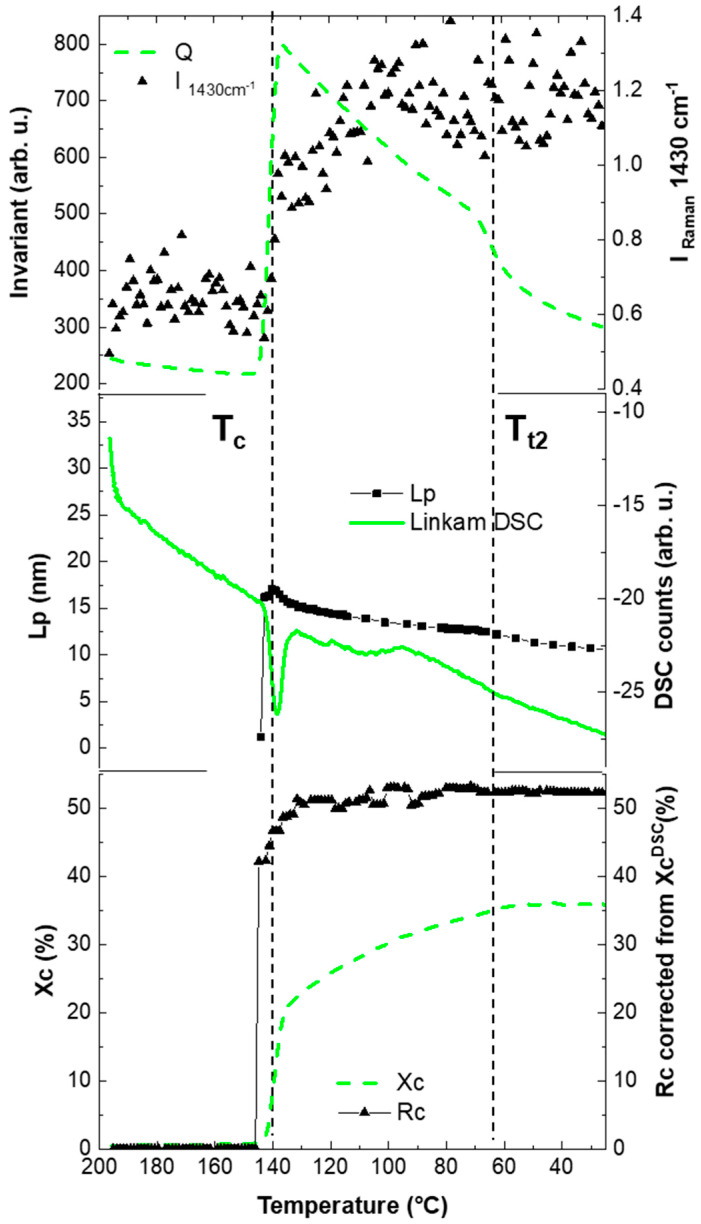
Porod invariant (Q), crystallinity (X_c_) and long period (L_p_) evolution as a function of temperature during crystallization from the melt (200 °C) of PVDF derivative. The dashed line (≈145 °C) indicates the onset of crystallization transition (T_c_) and the dashed line (≈65 °C) the transition of the secondary crystallization/rigid amorphous compaction (T_t2_).

Likewise, Raman spectroscopy is known to be sensitive to the phase variation of PVDF (α/β ratio) [68]. In particular, the vibrational bands at 796 and 840 cm^−1^ (see Figure 5C) of the α-phase [83,84,85] correspond to the rocking vibration mode r (CH_2_)-νs. (CF_2_) and r (CF_3_) [86], respectively, were monitored as a function of temperature (see Figure 6) to follow the PVDF crystallization by Raman. Initially, at the melt (200 °C) a broader band was found that was slightly shifted to the α-phase, centred at 799 cm^−1^, corresponding to the rocking vibration mode of the amorphous phase of the PVDF (see Figure 5C).

However, as the temperature decreased, the bandwidth decreased due to the standstill of the vibration group r (CH_2_)-νs. (CF_2_) related to the amorphous to crystalline phase transition. Furthermore, the α-phase band of the blend (PVDF/fluoroelastomer) associated with the chain conformation $\left(\mathrm{TGT}\bar{G}\right)$ of the crystalline monoclinic phase (pseudo-orthorhombic) remained unchanged during the crystallization, confirming the β-phase absence revealed by the WAXS. Moreover, the integrated value of the characteristic α-vibration band (785–815 cm^−1^) was normalized with the offline DSC-Raman data (see Figure S3) to assign apparent crystallinity content to the Raman spectra, independently from the X-rays experiments. The nanostructure parameters of the two-phase lamellar system with diffuse boundaries was evaluated using the correlation function (see Figure 5D) to understand the mechanism of crystallization from the scattering data. The evolution of the long period, crystallinity content, lamellar thickness and molecular conformation as a function of temperature during the crystallization from the melt of the blend PVDF/fluoroelastomer was determined (see Figure 6).

The slow increase in the α-vibration band before Tc, which was not evident in any of the parameters derived from the X-ray techniques (Lp, Q and Xc), suggested the previous arrangement of the molecular chain to induce the crystallization. In addition, the thermal behaviour of the Raman reference band for spectra intensity normalization (centred at 1430 cm^−1^) was assessed to correlate with macroscopic phase changes. The Raman reference band should be constant upon thermal changes as belongs to a vibration band of a molecular group unrelated to the main polymeric chain backbone of the amorphous region. Likewise, the integration of the band centred at 1430 cm^−1^ was sensitive to the phase changes as a result of the scattering nature of Raman spectroscopy. Indeed, the dramatic increase during the first crystallization and the subsequent stabilization indicated the rapid crystalline formation across the sample upon cooling at different length-scales (see Figure 6).

Moreover, the crystallization temperature observed by Raman spectroscopy (changes in the 796 cm^−1^ band) was approximately 139± °C, confirming the value detected by the Linkam DSC (138.7± °C) (see Figure 6).

Furthermore, the SAXS analysis of the isotropic two-phase system [87], as an approximate system for the blend PVDF/fluoroelastomer (amorphous and crystalline phase) was achieved by using the Porod invariant, *Q*, (Equation (S4) in Supplementary Materials) [88].

The invariant evolution as a function of temperature (see Figure 6) shows the phase transition at 140 °C that corresponds to PVDF crystallization. At the moment of crystallization, a two-phase system was formed; the amorphous part of the solidified material featured approximately the same electron density, but the electron density of the crystalline phase was higher. The maximum indicated that 50% of the material was in the crystalline phase. However, there was a discrepancy between the crystallinity detected at the inflexion point as well as the end of the crystallization process by both Raman and WAXS. Moreover, a discrepancy in the crystallinity content was found by Raman (calibrated by the DSC-Raman) and WAXS, which was potentially associated with an underestimation of the fitting of the crystalline portion.

Besides the final crystallinity content, the measured crystallization kinetics showed different trends between the three techniques, reflecting the different experimental sensitivities. The sudden drop of the invariant right after the crystallization onset, together with the decrease of the Lp, contradicted the continuous slow crystallization increase upon cooling observed by WAXS, indicating the formation of intra-lamellar structures, assuming a two-phase model with fixed electron densities. Similarly, a further decrease in both the invariant and the Lp at around 65 °C (inflexion point determined by derivative), together with the negligible increase in crystallinity (see Xc from WAXS in Figure 6), suggested the onset of a secondary crystallization or rigid amorphous compaction (designed as transition temperature 2, T_t2_, in Figure 6).

### 3.2. Correlation Data: Chemometrics

The data mining was facilitated by chemometrics methods, especially the employment of a data fusion/multiblock analysis approach to correlate the simultaneously acquired data. The large amount of data generated (see Figure S4) with subtle changes via a multi-transition of the structured system generally observed by indirect methods required extensive analysis by mathematical tools to highlight slight data changes. The PCA is a reduction method of the original data using an orthogonal transformation to convert the data into linearly uncorrelated data, the so-called principal component (PC) to represent the largest variance possible [89]. A great advantage of PCA analysis is the ability that offers to extract data separately from noise. The signal extraction can even be achieved with data with increased signal-to-noise levels, which is quite often the case with time-resolved data, where the evolution of the system determines the time-frame length instead of allowing to collect every individual frame long enough to achieve high data quality. Furthermore, the application of the PCA to the multidataset fused by a SUM-PCA [69] permitted us to transform the different measured variables into a combined data matrix that contained all the relationships between crystallinity, long period and chain conformation, which is related to the vibrational mode, as a function of temperature (see Figure S5).

**Figure 7:**
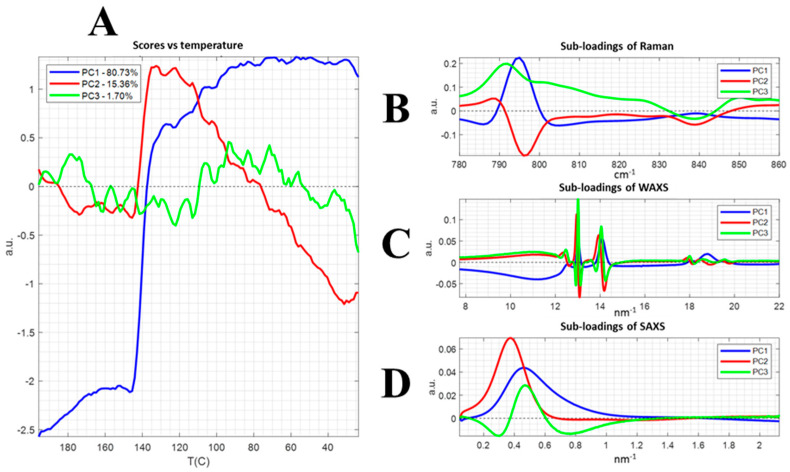
Multiblock PCA. Block-scaled and normalized (mean-centred) PCA model, with the scores as a function of temperature (**A**), the sub-loading of Raman (**B**), WAXS (**C**) and SAXS (**D**).

The data matrix was analyzed by either standard PCA or the SUM-PCA to obtain the scores (i.e., the time evolution of different principal components) and loadings (i.e., the spectral/scattering profiles differences of each principal component) of each data set or the block-normalized combined data, respectively. The scores correspond to the weights of the spectra/scattering profiles in the different dimensions (PC), while the loadings represented the coefficients of the linear combination of the initial variables (herein, the Raman band, or the scattering vector q), as well as indicating the variance percentage attributed to each PC. Both PCA and SUM-PCA (see Figure 7 for SUM-PCA to show the correlation between the techniques) were applied to the acquired data of every technique of the combined setup (Raman spectra, SAXS and WAXS) during the crystallization from the melt to room temperature as the crystallization event covered the entire vector variance.

Similarly, the scores represent the temperature evolution of each of the three processes retrieved by the SUM-PCA that conveyed the phase transformation from the melt (see Figure 7A). Besides, the loadings depicted the involved variables of each of the processes that occurred during the phase transition analyzed in common. The PC was interpreted by relatively associating the loading value and, thus, negative values of the score were correlated with negative loading values; the same approach was used for the positive score values with the corresponding positive loadings.

The SUM-PCA disclosed that the structural transformation was divided into three different processes related to different rates of crystallization and possibly different crystalline domains. Likewise, the PC1 correspond to the most important process that described 80% of the variance and was related to the main amorphous-to-crystalline phase transformation (inflexion point at the crystallization temperature), whereas the PC2 indicated a concomitant rearrangement of the polymeric chains, as well as the packing and growth of the crystalline phase, following a trend similar to the invariant (see Figure 6). In addition, the temperature evolution of the PC3 suggested an association with the weak transitions that occurred at 100 °C and 60 °C weights (see Figure 6), which was in agreement with the 1.7% of the variance of the system transformation.

In particular, the loadings of the PCA highlighted the importance of slight changes, such as the Raman vibration band at 840 cm^−1^ and characteristics of the vibration mode r (CH_2_) that were correlated to the crystalline domains by other techniques, as well as the association with the transition that occurred at 65 °C.

**Figure 8:**
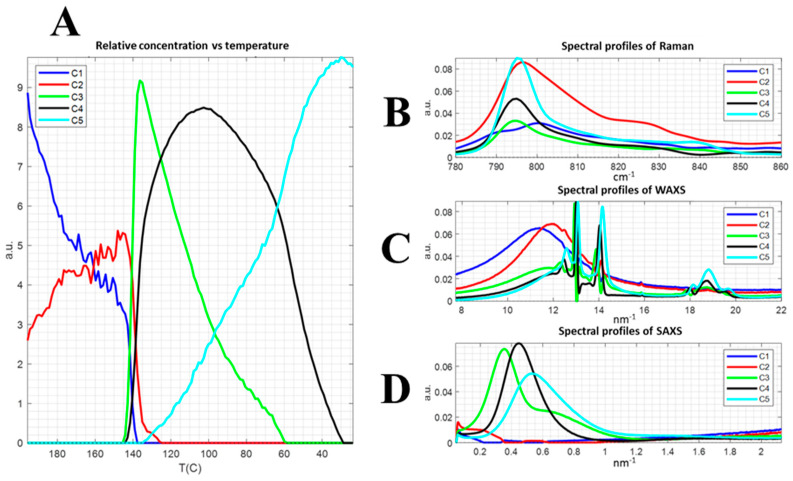
Multiblock MCR. Normalized spectra. Five components. Unimodality in C. Nonnegativity in C and S. The relative concentration as a function of temperature (**A**), the spectral profiles of Raman (**B**), WAXS (**C**) and SAXS (**D**).

The MCR-ALS (Multivariate Resolution of Curves) was applied to decompose without a priori assumptions to a set of data of the single components, herein the Raman spectra as well as the scattering profiles (see Figure 8) [90]. The MCR-ALS is one of the only methods with which it is possible to apply constraints, such as the non-negativity of spectra/profiles and concentrations since the negative values of the WAXS profile, SAXS profile and Raman spectrum are physically meaningless. The MCR can assist in the identification of ambiguous or weak data, as well as data that are made difficult to assign by the structural complexity of polymers. Similarly, the MCR manifested the importance of the Raman band centred around 840 cm^−1^ associated with the organization of the head-to-head and tail-to-tail chain defects, as well as its concentration increase during the transition at 65 °C, which is controversially identified in previous research.

The variables components extracted by MCR-ALS manifested the packing of the amorphous phase (C1 and C2) in the melt during the cooling step before the crystallization onset (C3). The concentration evolution of C3 with temperature followed the same trend that the SAXS invariant (see Figure 6). In addition, the C4 components related to the crystalline phase, as well as the concentration tendency with temperature, indicated the crystallization at a different rate of a different domain. Furthermore, the C5 represented the phase transformation of a less defined crystalline domain, as shown by the SAXS and WAXS components, which evolved with an inflexion point at the transition temperature of weak phase transformation at 65 °C in agreement with PC3.

The chemometric analysis of the data set fusion of the multi-technique approach enabled us to correlate both the correlations of the variables and their temporal evolutions of more than two techniques compared to previous homospectral and heterospectral 2DCOS [58,60]. In particular, it allowed us to confirm the continuous broad crystallization thermal range slightly observed by MDSC (low enthalpic transition) by the techniques correlation, as well as to identify the chemical nature of the debated transition at 65 °C. However, an in-depth discussion of the correlation between the different data sets and the importance of subtle data changes is beyond the scope of this paper and further DSC characterization will be performed to support our initial findings. This will be described in a future publication.

## 4. Conclusions

The combination of Raman spectroscopy with simultaneous SAXS/WAXS experiments was reviewed, emphasizing the experimental preventive measurements to match thermodynamical conditions observed by each technique. In particular, by concomitantly probing the phase transformations, the synergetic analysts enables us to discard likely detrimental X-ray side effects on the kinetics and chemical transformation of the material under study. The impact of the selected technical elements on the data quality, as well as on the chemical information accessible through the measurement conditions, was also discussed. The enlargement of the length-scale under study by Raman spectroscopy uncovered the masked interrelationship of the hierarchical structural organization during the phase transformation. In particular, low energetic phase transformations undetected by standard DSC are associated with a weak structural reorganization that required more sensible technics (MDSC) capable of distinguishing complex thermodynamic events. Moreover, the multivariate analysis applied to the multidataset fusion could potentially unburden the data analysis task of tedious data processing and fitting routines, as well as correlating the variables under study. In particular, the study by chemometric analysis of fused multidatasets from different techniques (more than two) permits the interpretation of unclear data variables by learning from the correlation with other variables of the combined setup that enrich the overall explanation of the phenomena. Herein, the detection of the presence of a low-intensity band in the Raman spectra by chemometric methods that was difficult to be assigned and was previously correlated with the crystallization of the head-to-head and tail-to-tail chain defects, was key to understanding the nature of the controversial slight thermal transition that occurred around 65 °C.

The detailed analysis of the PCA and MCR methods of the phase transformations, as well as their development, was found to extract similar information to the typical structural analysis of the polymers by scattering techniques.

In particular, the “scores” (weighting coefficients) of PC1 and PC2 for each measurement technique illustrated the weight of similar variations within the Raman spectra and SAXS/WAXS profiles according to the definitions of the “loadings” or PCs. Interestingly, the study of the trend of the score yielded an equivalent pattern evolution for each technique in the new representation space, which was composed of PC1, PC2 and PC3, although the techniques were sensitive to different structural parameters at different scales. In addition, the MCR analysis enable us to decompose the main components of the system and their individual evolutions, as well as the temporal correlation among them, without a priori data knowledge. The application of chemometric analysis to the polymeric field through multiple compatible techniques offers the possibility to learn the identification of complex data that could potentially assist in the development of methods to transfer data interpretation to techniques able to monitor polymer processing in industrial environments.

## Supplementary Materials

The following are available online at https://www.mdpi.com/article/10.3390/polym13234203/s1. Figure S1: Calibration curve obtained under the simultaneous influence of both X-ray beam and Raman laser (785 nm) measured by the Linkam DSC600-stage. The straight line indicates the measured data and the blue dotted curve is the ‘ideal’ line where no systematic error would be present. Only a slight deviation at elevated temperatures is observed; Figure S2: DSC standalone-Raman setup (A) and the acquired 3D plot Raman spectra obtained during the cooling step from the melt to RT (B); Figure S3: Fitting of the Raman intensity acquired on the simultaneous SAXS/WAXS setup com-bined to both Linkam DSC and Raman spectroscopy techniques of the deconvoluted vibrational bands. The Gaussian functions (G1, G2 and G3) correspond to the amorphous phase whilst Lorentz function (lor) were selected to adjust the Raman bands associated with the crystalline chains (A). The crystalline ratio was calculated from the Raman spectra (Rc) and calibrated with the crystalline content obtained (Xc) by WAXS (B). First, the signal to noise ratio of the two methods has been compared. Similar data quality was obtained apart from a smoother profile in the DSC-Raman spectra due to the lower background. The lower background is related to the com-pletely closed environment enabled by the smaller waist dimension of the laser spot size (2*W*_0_ = 5.8 μm and 2*Z_R_* = 0.19 mm) due to the shorter focal distance in the DSC-Raman setup (1.5 cm); Figure S4: Raw data of the PVDF/fluorelastomer blend derivative for Raman (A), WAXS (B) and SAXS (C) techniques upon crystallization from the melt; Figure S5: MVA matrix explanation: row-wise arrangement (A), SUM-PCA (B) and MCR (C).
