# Supplementary material for: Data Mining of Polymer Phase Transitions upon Temperature Changes by Small and Wide-Angle X-ray Scattering Combined with Raman Spectroscopy
Source: Polymers (Basel). 2021 Nov 30;13(23):4203. doi: 10.3390/polym13234203 (PMC8659756; doi:10.3390/polym13234203)
Supplement: Supplementary file 1 [file polymers-13-04203-s001.zip › polymers-1458601-supplementary.pdf]

polymers

MDPI

Data Mining of Polymer Phase Transitions upon Temperature Changes by Small and Wide-Angle X-Ray Scattering Combined with Raman Spectroscopy

Sarah Saidi [1,2], Giuseppe Portale [3], Wim Bras [2,4], Alessandro Longo [5,6], Jose M. Amigo [7,8], David Chapron [1], Patrice Bourson [1] and Daniel Hermida-Merino [1,2,*]

1 LMOPS, Université de Lorraine, CentraleSupelec, 2 rue Edouard Belin, 57070 Metz, France; saidi.sarah1@hotmail.fr (S.S.); david.chapron@univ-lorraine.fr (D.C.); patrice.bourson@univ-lorraine.fr (P.B.)
2 DUBBLE@ESRF, Netherlands Organization for Scientific Research (NWO), BP CS40220, 38043 Grenoble, France; brasw@ornl.gov
3 Macromolecular Chemistry & New Polymeric Materials, Zernike Institute for Advanced Materials, University of Groningen, Nijenborgh 4, 9747 AG Groningen, The Netherlands; g.portale@rug.nl
4 Oak Ridge National Laboratory, Chemistry Division, Oak Ridge, TN 37831, USA
5 ID20, ESRF, 71 Avenue des Martyrs, 38000 Grenoble, France; alessandro.longo@esrf.fr
6 Istituto per lo Studio dei Materiali Nanostrutturati (ISMN)-CNR, UOS Palermo, Via Ugo La Malfa, 153, 90146 Palermo, Italy
7 IKERBASQUE, Basque Foundation for Science, 48011 Bilbao, Spain; josemanuel.amigo@ehu.eus
8 Department of Analytical Chemistry, University of the Basque Country UPV/EHU, P.O. Box 644, 48080 Bilbao, Spain
* Correspondence: daniel.hermida-merino@esrf.fr

Citation: Saidi, S.; Portale, G.; Bras, W.; Longo, A.; Amigo, J.M.; Chapron, D.; Bourson, P.; Hermida-Merino, D. Data Mining of Polymer Phase Transitions upon Temperature Changes by Small and Wide-Angle X-Ray Scattering Combined with Raman Spectroscopy. *Polymers* **2021**, *13*, 4203. https://doi.org/10.3390/polym13234203

Academic Editor: Ki Hyun Bae

Received: 25 October 2021
Accepted: 26 November 2021
Published: 30 November 2021

Publisher's Note: MDPI stays neutral with regard to jurisdictional claims in published maps and institutional affiliations.

Copyright: © 2021 by the authors. Licensee MDPI, Basel, Switzerland. This article is an open access article distributed under the terms and conditions of the Creative Commons Attribution (CC BY) license (https://creativecommons.org/licenses/by/4.0/).

Abstract: The complex physical transformations of polymers upon external thermodynamic changes are related to the molecular length of the polymer and its associated multifaceted energetic balance. The understanding of subtle transitions or multistep phase transformation requires real time phenomenological studies using a multi-technique approach that covers several length-scales and chemical states. A combination of X-ray scattering techniques with Raman spectroscopy and Differential Scanning Calorimetry has been conducted to correlate the structural changes from the conformational chain to the polymer crystal and mesoscale organization. The state of the art and the experimental combination of Raman spectroscopy with simultaneous SAXS/WAXS measurements couple to a DSC is discussed. In particular, we show that in order to obtain the maximum benefit from simultaneously obtaining high-quality data sets from different techniques, one should look beyond traditional analysis techniques and instead apply multivariate analysis. Data mining strategies can be applied to develop methods to control polymer processing in an industrial context. Crystallization studies of a PVDF blend with a fluoroelastomer, known to have complex phase transitions, were used to validate the combined approach and further analyzed by MVA.

Keywords: Polymer; X-ray scattering; Raman spectroscopy; Combined techniques; Multivariate analysis.

Supplementary Materials

1. Materials and Methods

1.1. In-situ X-ray scattering

Time-resolved simultaneous small-angle X-ray scattering (SAXS) and wide-angle X-ray scattering (WAXS) experiments (X-ray wavelength = 0.1 nm), were performed on the European Synchrotron Radiation Facility (ESRF), at DUBBLE (BM26B, Grenoble, France)

[1,2]. Acquisition of simultaneous SAXS and WAXS patterns with an exposure time of 15 seconds was collected with a Pilatus 1M detector [3,4] at a distance of 3.5 m from the sample cell) and a Pilatus 300K detector (1472 × 195 pixels of 172 μm × 172 μm placed at a distance of 0.31 m), respectively. The beam size was around 300 μm × 300 μm and its intensity on the sample was ca 10^{11} photons/s. The data was corrected from background contributions and normalized for synchrotron source intensity decay and X-ray transmission. AgBe (silver behenate) and α-Al2O3 (alumina) were used to calibrate the scattering vector q-scale, where q = 4πsinθ/λ with θ being half of the scattering angle.

Data reduction of SAXS/WAXS data was performed with bubble [5] as the data integration software to obtain 1D intensity profiles as a function of the scattering vector (q = 4πsinθ/λ) and expressed in arbitrary units for both SAXS and WAXS profiles. The deconvolution of the 1D WAXS profile between 8 to 22 nm^{-1} using Lorentzian functions for the reflections (100), (020), (110), and (021) of the PVDF α-phase and a pseudo-Voigt function for the amorphous halo has been conducted to calculate the relative degree of crystallinity (X_c) by equation (S1):

$$X_c = \frac{A_{crystalline}}{A_{crystalline}+A_{amorphous}} \times 100 \qquad \text{(S1)}$$

A first estimation of the long period (L_p) value was determined from the Lorentz corrected 1D SAXS intensity profiles applying the relationship: $L_p = 2\pi/q_{max}$, where q_{max} is the value of the scattering vector corresponding to the maximum of the correlation peak of the integrated intensity. Typically, the maximum of the Lorentz-correct SAXS reflection is associated with the approximately long period of a periodic lamellar structure of a semi-crystalline polymer by:

$$L_{pB} = \frac{2\pi}{q_{max}} \qquad \text{(S2)}$$

Moreover, a detailed analysis of the nanostructure of the fluoroelastomer/PVDF blends has been conducted applying the 1D correlation function, $\Gamma_1(x)$, of a lamellar system using the SasView software (http://www.sasview.org/).

$$\Gamma_1(x) = \frac{1}{Invariant}\int_0^{\infty} I(Q)Q^2\cos(Qx)\,dQ \qquad \text{(S3)}$$

Where the invariant can be expressed by the given equation (S4) assuming a biphasic system:

$$Q = \int_0^{\infty} q^2 I(q)\,dq \;\propto\; 2\pi\Phi\,(1-\Phi)(\rho_c-\rho_a)^2 \qquad \text{(S4)}$$

Where I(q) is the scattering intensity and q the scattering vector. In the case of the second term of the equation (S4), Φ and ρ_a are the volume fraction and the electron density of the amorphous phase, respectively, while (1- Φ) and ρ_c are the volume fraction and the electron density of the crystalline phase respectively.

The temperature control was performed using a Linkam DSC 600 cell [6], applying a heating and cooling rate of 5 °C/min, starting from room temperature (RT) to 200 °C, where an isothermal treatment of 10 min has been conducted to erase the material thermal history.

1.2. Calibration of the Temperature from the linkam hot stage

The melting point of different well-known standards (Nylon, indium, benzoic acid, PVDF) has been used to cover the linearity response of the instrument in the temperature range under interest (see calibration curve in Figure S1), measurements conducted before the SAXS/WAXS/DSC/Raman beamtime measurement.

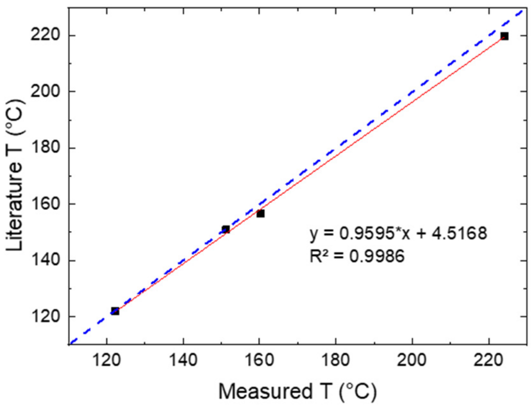

Figure S1. Calibration curve obtained under the simultaneous influence of both X-ray beam and Raman laser (785 nm) measured by the Linkam DSC600-stage. The straight line indicates the measured data and the blue dotted curve is the 'ideal' line where no systematic error would be present. Only a slight deviation at elevated temperatures is observed.

1.3. Differential Scanning Calorimetry

The sample thermal behaviour was determined using a DSC TA instrument Q200 apparatus. A DSC combined with Raman spectroscopy (Rxn1 Kaiser Optical Systems, 785nm) has been also conducted with a heating/cooling rate of 5 °C/min with thermal protocol cycles from room temperature (RT) to 200 °C and back to RT. The Raman spectrometer parameters duplicate the experimental conditions of the previous Raman measurements with the SAXS/WAXS/DSC setup apart from the focal distance where an adjustable MR probe (Kaiser) focal lens was set to 1.5 cm directly to the DSC heating element using open sample pans[7].

Likewise, the temperature recorded in the header of every single SAXS and WAXS frame as well as Raman spectra are calibrated this way. The quality of the in-situ Raman spectra acquired in the experimental coupled with X-ray scattering and Linkam DSC techniques has been compared with the data obtained by combining a standard DSC and the same Raman spectrometer (Figure S2), a measurement performed prior to the SAXS/WAXS/DSC/Raman beamtime measurement [7].

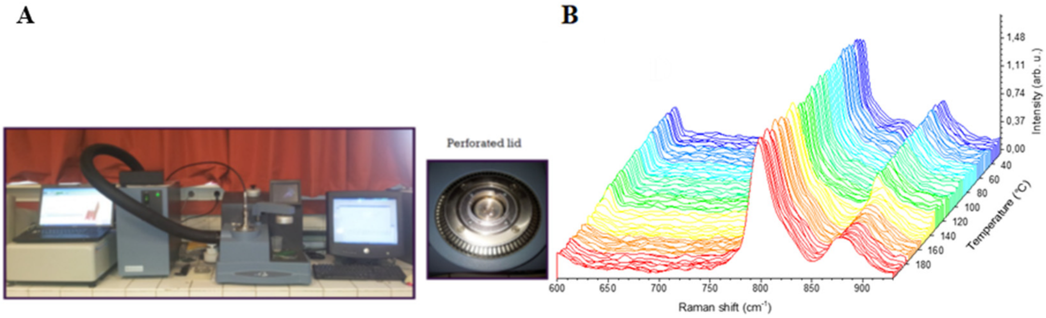

Figure S2. DSC standalone-Raman setup (A) and the acquired 3D plot Raman spectra obtained during the cooling step from the melt to RT (B).

The temperature calibration permits to amend the experimental error of the thermocouple reading to compare the material response in the different setups. Furthermore, X-rays damage effects are easily identified by the comparison of the Raman spectra collected on both Raman/DSC and Raman SAXS/WAXS/DSC setups.

1.4. Raman Spectroscopy

Raman spectroscopy in reflection mode was performed using an RXN1 spectrometer from Kaiser Optical Systems, a compact and transportable spectrometer with an integrated laser with an excitation wavelength of 785 nm. The laser wavelength and power (150 mW) is chosen to minimise sample fluorescence, heating and damage

The Raman Rxn1™ instrument is based on an f/1.8 axial transmissive spectrograph allowing non-contact detection with a spectral resolution of 2 cm^{-1} For these experiments the Raman probe was fitted with a lens with a focal distance of 5 cm to obtain both good signal/noise ratio as well as a suitable beamspot size to prevent interference with the SAXS/WAXS experiments. Raman spectra acquisition were synchronized with the SAXS/WAXS images by acquiring spectra of 10 s (good signal to noise ratio) with a 5 s sleeping time in between spectra, recording a spectrum every 1.25 °C. The position of the Raman probe can be adjusted for direction and focal plane using an XYZ table.

The acquired Raman spectra have been analysed using the iCRaman™ (Mettler Toledo) software. The spectra baseline has been corrected and normalized for reflectivity variations and volume changes using as a reference the stable Raman band centred at 1430 cm^{-1} [8].

The Raman spectra have been decomposed by a MATLAB script, using a Lorentz function (lor) to the band centred at 795 cm^{-1} that is characteristic of the rocking vibrational mode (CH_2) - ν_s (CF_2) corresponding to the PVDF α phase. Furthermore, the amorphous content has been adjusted to the Gaussian functions (G1, G2 and G3) located between 797 and 800 cm^{-1} for the G1 band associated to (CH_2) - ν_s (CF_2), 814 and 820 cm^{-1} for G2 and finally, between 829 and 840 cm^{-1} for G3 band linked to the vibrational band either of the rocking of (CH_2) PVDF or the rocking of the fluoroelastomer (CF_3) group. The crystallinity ratio (R_C) detected by Raman spectroscopy has been determined from the ratio between either the area or the intensity of the vibration bands decomposed previously (see Figure S3 and equation S5). However, the Raman vibration band intensity has been found to enhance the robustness of the crystallinity ratio analysis since it is less temperature dependent.

$$R_c = \frac{I_{lor}}{(I_{lor}+I_{G1}+I_{G2}+I_{G3})} \quad \text{(S5)}$$

The crystallinity ratio has been cross correlated with the crystalline content obtained by WAXS in order to remove ambiguities due to variations in the volume that was illuminated by the Raman laser (see Figure S3B).

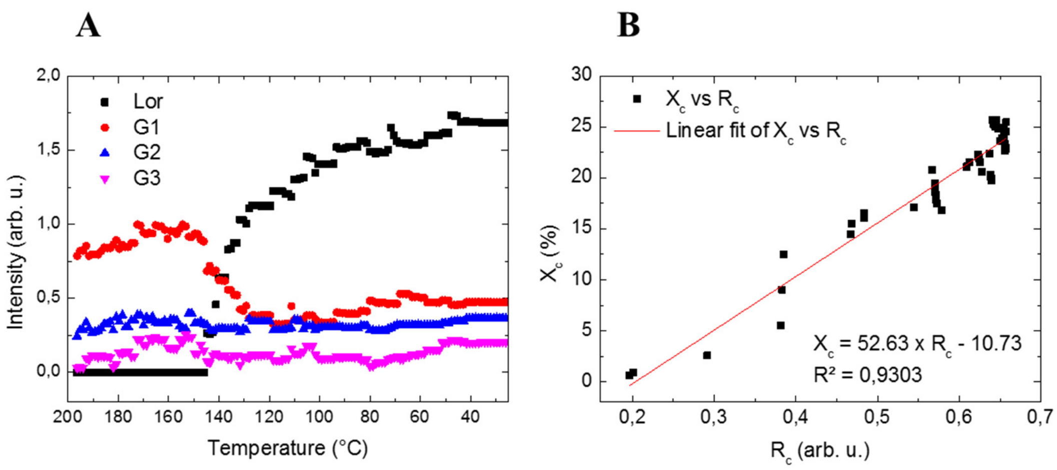

Figure S3. Fitting of the Raman intensity acquired on the simultaneous SAXS/WAXS setup combined to both Linkam DSC and Raman spectroscopy techniques of the deconvoluted vibrational bands. The Gaussian functions (G1, G2 and G3) correspond to the amorphous phase whilst Lorentz

function (lor) were selected to adjust the Raman bands associated with the crystalline chains (A). The crystalline ratio was calculated from the Raman spectra (R_c) and calibrated with the crystalline content obtained (X_c) by WAXS (B). First, the signal to noise ratio of the two methods has been compared. Similar data quality was obtained apart from a smoother profile in the DSC-Raman spectra due to the lower background. The lower background is related to the completely closed environment enabled by the smaller waist dimension of the laser spot size ($2W_0$ = 5.8 μm and $2Z_R$ = 0.19 mm) due to the shorter focal distance in the DSC-Raman setup (1.5 cm).

2. Multivariate analysis: detailed methodology

2.1. Arrangement of the data for data fusion/multiblock analysis

First, the raw data is arranged for data fusion/multiblock analysis to correlate the physicochemical variables detected by the different techniques. Each technique provides a set of raw data that was arranged as three independent matrices, one for each method (see Figure S4 and S5). The temperature corresponds to the row direction for every matrix, being the common mode for all three experiments; while the spectral pattern is placed in the column direction and is different for each experiment.

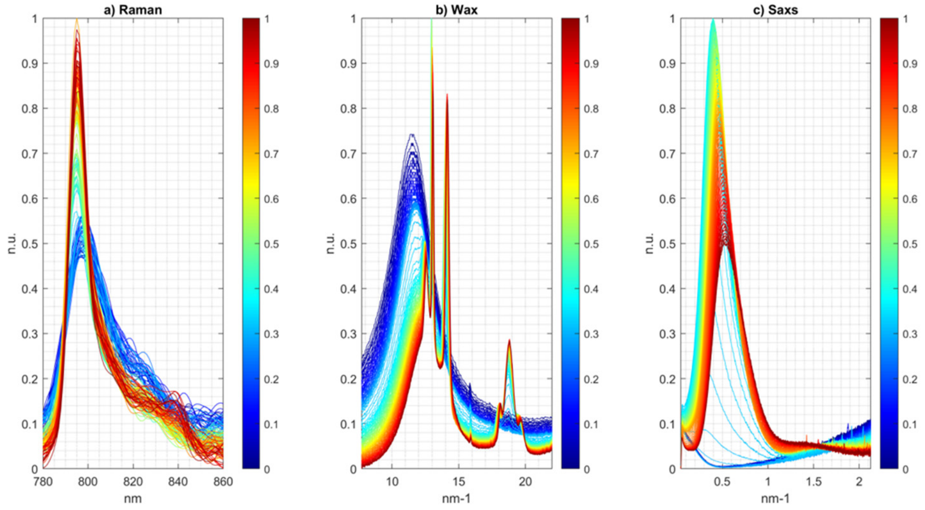

Figure S4. Raw data of the PVDF/fluorelastomer blend derivative for Raman (A), WAXS (B) and SAXS (C) techniques upon crystallization from the melt.

Likewise, the Raman matrix, $\mathbf{X}_R$, is represented by the following dimensions (134 x 81) whilst the WAXS matrix, $\mathbf{X}_W$. features the dimensions (134 x 906), and the SAXS matrix, $\mathbf{X}_S$, has the dimensions (134 x 1301) (see Figure S5A).

The most straightforward manner of fusing the three matrices to be analysed together is by the so-called row-wise augmentation. However, the block data must be normalised before the data combination for augmenting the block. The block-normalisation scales every block by the square root of the pooled variance of its corresponding variables to ensure that the data blocks contribute equally to the pooled variance of the final model. The final matrix **X** will be formed as indicated in equation (S6):

$$\mathbf{X} = [\ \mathbf{X}_{Rb}\ \mathbf{X}_{Wb}\ \mathbf{X}_{Sb}] \qquad \text{(S6)}$$

Where **X** will have dimensions (134 x 2288). The subindex b in equation (6) indicates the block-normalized data (Figure S5A).

2.2. SUM-PCA

The SUM-PCA is based on applying the standard PCA on the matrix formed by the augmented blocks [9,10]. Therefore, SUM-PCA will decompose the **X** matrix (after mean centring) into two submatrices as indicated in equation (S7) (Figure S5B):

$$\mathbf{X_n} = \mathbf{T_{sup}} * \mathbf{P^T_{sup}} + \mathbf{E}_X \qquad (S7)$$

Where $\mathbf{X_n}$ denotes the mean-centred **X** matrix, $\mathbf{T_{sup}}$ contains the so-called super-scores (common scores to the three blocks) with the dimensions (134 x *F*), with *F* the appropriate number of Principal Components explaining all relevant variance of the combined blocks. $\mathbf{P^T_{sup}}$ is the so-called super-loadings, featuring dimensions (*F* x 2288). In SUM-PCA, the super-scores are constrained to be orthonormal, while the super-loadings are forced to be orthogonal. As in a normal PCA, the variance capture is referred to the amount of variance that each super Principal Component explains to respect to the matrix of the raw data **X**. The super-loadings matrix can be divided into each sub-loading matrix for each block, as indicated in equation S8:

$$\mathbf{P^T_{sup}} = [\mathbf{P^T}_R \ \mathbf{P^T}_W \ \mathbf{P^T}_S] \qquad (S8)$$

Where $\mathbf{P^T}_R$, $\mathbf{P^T}_W$, $\mathbf{P^T}_S$ are the so-called sub-loading matrices, featuring dimensions (*F* x 81), (*F* x 906) and (*F* x 1301), respectively. As an important note, while the super-loadings are orthogonal to each other, the individual sub-loadings for each block might not fulfil this constraint.

2.3. Multiblock-MCR

Multivariate Curve Resolution (MCR) aims at obtaining the underlying physicochemical behaviour of the data under study without assuming an a priori behaviour of the data (i.e. any empirical model is assumed) by only using the linear dependency between the concentration and the intensity of the measured signal [11,12]. Analogously to PCA, MCR will decompose the row-wise augmented matrix **X** into a subset of two submatrices as indicated in equation (S9) (Figure S5C):

$$\mathbf{X} = \mathbf{C_{sup}} * \mathbf{S^T_{sup}} + \mathbf{E}_X \qquad (S9)$$

Where **X** is the block scaled matrix containing the three data blocks. Where $\mathbf{C_{sup}}$ are the so-called concentration profiles, with dimensions (134 x *M*) and $\mathbf{S^T_{sup}}$ the pure spectral profiles with dimensions (*M* x 2288). One of the most common algorithms to perform the decomposition is the alternating least squares (ALS). Even being similar to the equation (S7), MCR is calculated without imposing orthogonality or orthonormality in the decomposition. Instead, $\mathbf{C_{sup}}$ and $\mathbf{S^T_{sup}}$ are calculated assuming their linear dependency and imposing soft constraints related to the natural behaviour of the signals, in order to avoid ambiguities in the decomposition. For instance, in our case, non-negativity in the concentration as well as for the spectral profiles can be imposed since no concentration profiles, neither spectra contain negative values.

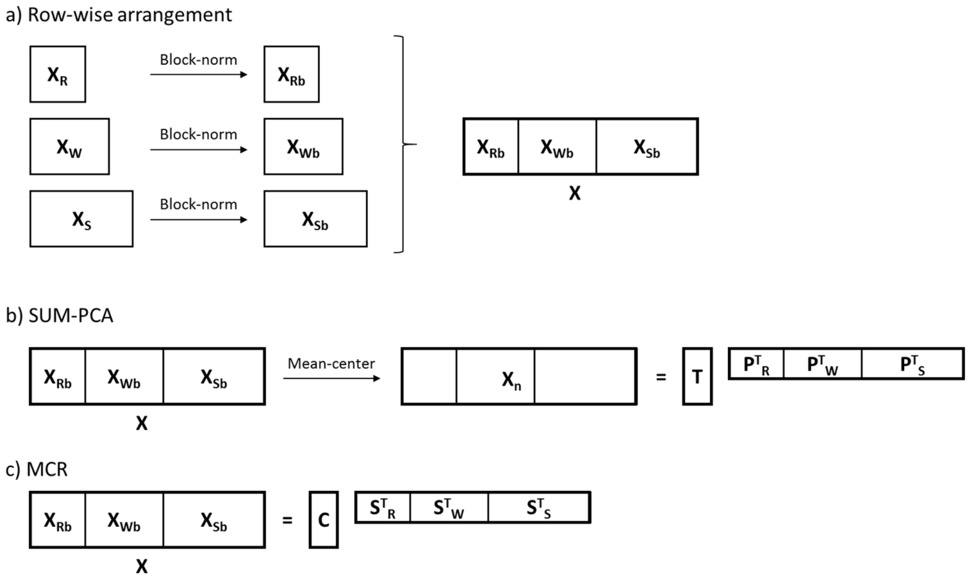

Figure S5. MVA matrix explanation: row-wise arrangement (A), SUM-PCA (B) and MCR (C).

Besides, the concentration profiles can be constrained to be unimodal, and thus, forced to possess only a maximum in the profile. When dealing with analysing several blocks simultaneously (multiblock), there is the possibility of constraining each block differently, so the physicochemical characteristics of each block may play an independent role in the spectral profiles while affecting the behaviour of the global concentration profiles. In our case, the three blocks were submitted to identical constraints.

One major difference with PCA is that in MCR the components calculated (M) are not constrained to be orthogonal. Instead, the MCR components must obey the general linear behaviour of the data, where the signal is linearly dependent on the concentration. Therefore, knowing the exact value of M is essential to obtain a reliable model either using the PCA or the physicochemical knowledge of the data. Moreover, since MCR is subjected to ambiguities, initial estimations are required to initiate the ALS iterations. The initial estimations can be of concentration profiles or spectral profiles of the M components. The closer to the final result the more reliable and less ambiguous model will be obtained.

References

1. Bras, W.; Dolbnya, I.P.; Detollenaere, D.; van Tol, R.; Malfois, M.; Greaves, G.N.; Ryan, A.J.; Heeley, E. Recent Experiments on a Small-Angle/Wide-Angle X-Ray Scattering Beam Line at the ESRF. *Journal of Applied Crystallography* **2003**, *36*, 791–794, doi:10.1107/s002188980300400x.
2. Portale, G.; Cavallo, D.; Alfonso, G.C.; Hermida-Merino, D.; van Drongelen, M.; Balzano, L.; Peters, G.; Goossens, J.; Bras, W. Polymer Crystallization Studies under Processing-Relevant Conditions at the SAXS/WAXS DUBBLE Beamline at the ESRF. *Journal of applied crystallography* **2013**, *46*, 1681–1689.
3. Donath, T.; Brandstetter, S.; Cibik, L.; Commichau, S.; Hofer, P.; Krumrey, M.; Lüthi, B.; Marggraf, S.; Müller, P.; Schneebeli, M.; et al. Characterization of the PILATUS Photon-Counting Pixel Detector for X-Ray Energies from 1.75 KeV to 60 KeV. *J. Phys.: Conf. Ser.* **2013**, *425*, 062001, doi:10.1088/1742-6596/425/6/062001.
4. Henrich, B.; Bergamaschi, A.; Broennimann, C.; Dinapoli, R.; Eikenberry, E.F.; Johnson, I.; Kobas, M.; Kraft, P.; Mozzanica, A.; Schmitt, B. PILATUS: A Single Photon Counting Pixel Detector for X-Ray Applications. *Nuclear Instruments and Methods in Physics Research Section A: Accelerators, Spectrometers, Detectors and Associated Equipment* **2009**, *607*, 247–249, doi:10.1016/j.nima.2009.03.200.
5. Dyadkin, V.; Pattison, P.; Dmitriev, V.; Chernyshov, D. A New Multipurpose Diffractometer PILATUS@SNBL. *Journal of Synchrotron Radiation* **2016**, *23*, 825–829, doi:10.1107/S1600577516002411.

6. Bras, W.; Derbyshire, G.E.; Bogg, D.; Cooke, J.; Elwell, M.J.; Komanschek, B.U.; Naylor, S.; Ryan, A.J. Simultaneous Studies of Reaction Kinetics and Structure Development in Polymer Processing. *Science* **1995**, *267*, 996–999, doi:10.1126/science.267.5200.996.
7. Veitmann, M.; Chapron, D.; Bizet, S.; Devisme, S.; Guilment, J.; Royaud, I.; Poncot, M.; Bourson, P. Thermal Behavior of PVDF/PMMA Blends by Differential Scanning Calorimetry and Vibrational Spectroscopies (Raman and Fourier-Transform Infrared). *Polymer Testing* **2015**, *48*, 120–124, doi:10.1016/j.polymertesting.2015.10.004.
8. Strobl, G.R.; Hagedorn, W. Raman Spectroscopic Method for Determining the Crystallinity of Polyethylene. *Journal of Polymer Science: Polymer Physics Edition* **1978**, *16*, 1181–1193.
9. Ríos-Reina, R.; Callejón, R.M.; Savorani, F.; Amigo, J.M.; Cocchi, M. Data Fusion Approaches in Spectroscopic Characterization and Classification of PDO Wine Vinegars. *Talanta* **2019**, *198*, 560–572, doi:10.1016/j.talanta.2019.01.100.
10. Cocchi, M. *Data Fusion Methodology and Applications*; Elsevier, 2019; ISBN 978-0-444-63985-1.
11. de Juan, A.; Tauler, R. Chemometrics Applied to Unravel Multicomponent Processes and Mixtures. *Analytica Chimica Acta* **2003**, *500*, 195–210, doi:10.1016/S0003-2670(03)00724-4.
12. Juan, A. de; Tauler, R. Multivariate Curve Resolution (MCR) from 2000: Progress in Concepts and Applications. *Critical Reviews in Analytical Chemistry* **2006**, *36*, 163–176, doi:10.1080/10408340600970005.
